# *Panax notoginseng*: Pharmacological Aspects and Toxicological Issues

**DOI:** 10.3390/nu16132120

**Published:** 2024-07-02

**Authors:** Cesare Mancuso

**Affiliations:** 1 Fondazione Policlinico Universitario A. Gemelli IRCCS, Largo F. Vito 1, 00168 Rome, Italy; cesare.mancuso@unicatt.it; Tel.: +39-06-30154367; Fax: +39-06-3050159; 2 Department of Healthcare Surveillance and Bioethics, Section of Pharmacology, Università Cattolica del Sacro Cuore, Largo F. Vito 1, 00168 Rome, Italy

**Keywords:** free radicals, ginsenosides, gut microbiota, herbal products, medicinal plants, saponins

## Abstract

Current evidence suggests a beneficial role of herbal products in free radical-induced diseases. *Panax notoginseng* (Burk.) F. H. Chen has long occupied a leading position in traditional Chinese medicine because of the ergogenic, nootropic, and antistress activities, although these properties are also acknowledged in the Western world. The goal of this paper is to review the pharmacological and toxicological properties of *P. notoginseng* and discuss its potential therapeutic effect. A literature search was carried out on Pubmed, Scopus, and the Cochrane Central Register of Controlled Trials databases. The following search terms were used: “notoginseng”, “gut microbiota”, “immune system”, “inflammation”, “cardiovascular system”, “central nervous system”, “metabolism”, “cancer”, and “toxicology”. Only peer-reviewed articles written in English, with the full text available, have been included. Preclinical evidence has unraveled the *P. notoginseng* pharmacological effects in immune-inflammatory, cardiovascular, central nervous system, metabolic, and neoplastic diseases by acting on several molecular targets. However, few clinical studies have confirmed the therapeutic properties of *P. notoginseng*, mainly as an adjuvant in the conventional treatment of cardiovascular disorders. Further clinical studies, which both confirm the efficacy of *P. notoginseng* in free radical-related diseases and delve into its toxicological aspects, are mandatory to broaden its therapeutic potential.

## 1. Introduction

The first evidence of the medical use of crude herbs dates back to the mists of time when intelligent animals noticed that some food plants modified particular body functions. Human cultures institutionalized these observations so that the consumption of botanicals has often served important cultural traditions [1]. Over the last 2–3 decades, however, the use of botanicals has overcome the limits of folk medicine, and several herbal products are currently used as add-on therapies for free radical-related diseases, such as neurodegenerative, immune-inflammatory and cardiovascular disorders, diabetes mellitus, and cancer [2,3,4,5,6]. In this regard, herb-derived compounds, e.g., curcumin (the main ingredient of *Curcuma longa Lynn.*), ferulic acid (very abundant in Chinese herbs, including *Angelica sinensis* or *dang gui*, *Cimicifuga foetida,* and *Ligusticum chuanxiong*), ginkgolides (the active constituents of *Ginkgo biloba*), and ginsenosides (bioactive compounds abundant in ginseng species) have been extensively studied for their ability to scavenge reactive oxygen species (ROS) and enhance the cell stress response [7,8,9,10].

Ginseng is among the most popular botanical medications in both Europe and the United States of America (USA) because of its antioxidant, ergogenic, nootropic, and antistress activities [8,11,12]. Botanical preparations of ginseng may result from several species of *Panax*, the most commonly used being *Panax ginseng* (*P. ginseng* or Korean ginseng) and *Panax quinquefolius* (*P. quinquefolius* or American ginseng) [8,13,14]. *Panax notoginseng* (Burk.) F. H. Chen (*P. notoginseng*, also known as Chinese ginseng or *Sanqi*), grown in Yunnan and Guangxi, has long held first place in the whole patent medicines market in China, and the market volume of the single species has exceeded CYE 10 billion [15]. Globally speaking, according to The Trade Vision LLC (Cheyenne, WY, USA), the top three supplier countries for *P. notoginseng* are China, Canada, and the USA, whereas the top three importing countries are the Philippines, the USA, and Vietnam [16]. Lastly, the Global Notoginseng Root Extract Market report foresees a linear increase in the *P. notoginseng* world market over the 2024–2031 period, mainly as a pharmaceutical, cosmetic, and health supplement [17].

Available in many pharmaceutical forms, *P. notoginseng* has been used for the treatment of immune-inflammatory, cardiovascular, central nervous system (CNS), metabolic, and neoplastic diseases [15]. However, some studies report significant interactions with commonly used drugs (e.g., anticoagulants and antiplatelet agents), thus raising concerns on drug–herb interactions [15]. The aim of this paper is to review the pharmacological properties of *P. notoginseng*, focusing on pharmacognosy, pharmacokinetics, pharmacodynamics, and clinical studies demonstrating its efficacy in important diseases. Data on the toxicology of *P. notoginseng* will also be provided and discussed.

A literature search was carried out on the Pubmed, Scopus, and the Cochrane Central Register of Controlled Trials databases. The following search terms were used: “notoginseng”, “gut microbiota”, “immune system”, “inflammation”, “cardiovascular system”, “central nervous system”, “metabolism”, “cancer”, and “toxicology”. Only peer-reviewed articles written in English, with the full text available, have been included.

## 2. Pharmacognosy of *P. notoginseng*

**Figure 1:**
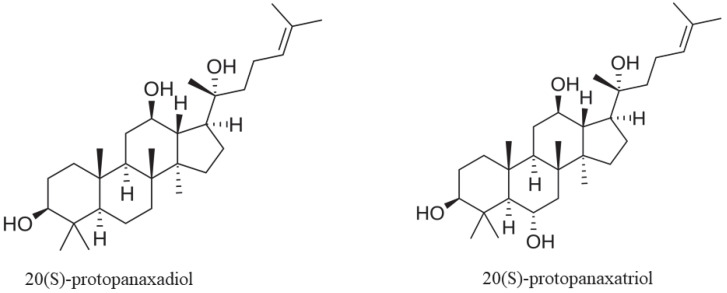
Chemical structures of both 20(S)-protopanaxadiol (PPD) and 20(S)-protopanaxatriol (PPT) saponins. The PPD group includes ginsenosides Rb1, Rb2, Rg3, Rc, and Rd, whereas the PPT group comprises ginsenosides Re, Rg1, Rg2, and Rh1. For further details, see Section 2.

More than 200 bioactive compounds have been identified in *P. notoginseng*, including saponins, flavonoids, cyclopeptides, sterols, polyacetylenes, volatile oils, amino acids, and polysaccharides [15]. Although these compounds are also common to both *P. ginseng* and *P. quinquefolius*, saponins, volatile oils (e.g., andrographolide), and polyacetylenes (e.g., panaxynol and panaxydol) are more abundant in *P. notoginseng* than in the other species [18]. Saponins, mainly dammarane triterpenes, are the principal bioactive compounds and include both 20(S)-protopanaxatriol (PPT)-derived ginsenosides and 20(S)-protopanaxadiol (PPD)-derived ginsenosides [8,15,19] (Figure 1). PPD-derived ginsenosides R1, Rb1, and Rd, together with PPT-derived ginsenosides Rg1 and Re, account for more than 90% of the total saponins in *P. notoginseng*, but they are not specific because they can be found in other species of the *Panax* genus [20,21,22]. However, among these saponins, ginsenosides Rb1 (30–36%), Rg1 (20–40%), and R1 (7–10%) are the most abundant and are currently considered as the standard compounds to evaluate the quality of *P. notoginseng* preparations [18,20,23]. Despite this differential composition, the total saponin content in *P. notoginseng* is much higher than in *P. ginseng* (~15% versus 4%, respectively), thus suggesting that, at the same dose, there is a greater concentration of bioactive compounds in the former than in the latter species [18]. *P. notoginseng* roots and rhizomes often undergo specific preservation processes, the most common being steaming. Notably, steaming results in the formation of novel ginsenosides, such as Rh4, Rk3, and Rg3, and this enrichment may be responsible for the specific pharmacological properties of steamed *P. notoginseng* [24,25]. Indeed, according to the current literature, steamed *P. notoginseng* is beneficial in patients with hematopoietic and immune diseases, whereas raw *P. notoginseng* counteracts swelling, eases pain, and improves lipid metabolism [24,26]. Interestingly, the ginsenoside composition in *P. notoginseng* may also vary depending on geographical distribution and climate factors. As shown by Liu et al., high annual precipitation and elevated humidity increase the content of ginsenosides R1, Rg1, and Rb1 [27].

## 3. Pharmacokinetics of *P. notoginseng*

After oral administration, *P. notoginseng* is metabolized by the gut microbiota through deglycosylation and dehydration reactions [28,29]. *Eubacterium*-, *Bifidobacterium*-, and *Streptococcus*-mediated deglycosylations are responsible for transforming the ginsenosides Rb1, Rd, Rc, and F2 into 20-O-β-D-glu-copyranosyl-20(S)-PPD, also known as compound K, which is the main metabolite with pharmacological effects [28,30,31]. Furthermore, through *Eubacterium*-, *Bifidobacterium*-, *Fusobacterium*-, and *Bacteroides*-mediated deglycosylation, the ginsenosides Rg1 and Re are converted into Rh1 and F1 [28,31].

Twelve male and twelve female healthy volunteers were treated with a single oral dose of *P. notoginseng* (90–270 mL of a water extract containing mainly ginsenosides Rg1, Rb1, Rd, R1, Re, Rh1, in decreasing concentrations) [32]. At the highest dose, the most abundant saponin detected in the plasma was Rb1, which reached peak plasma concentrations (C_max_) of 21–35 nM within 8–12 h and was excreted with a half-life (T_1/2_) of 33–57 h [32]. Importantly, compound K, absent in the ingested Sanqi extract, appeared in the plasma with a T_max_ (the time necessary to reach C_max_) of 11–17 h and exhibited much greater bioavailability than the parent Rb1 [area under the curve_0-t_ (AUC_0-t_) 4.75 µM∙h and 0.75 µM∙h, respectively] [32]. The relatively higher bioavailability of compound K in Chinese women than in men may be explained by the prevalence of the *Firmicutes* and *Actinobacter phyla*, which include *Eubacteria* and *Bifidobacteria*, the *genera* involved in ginsenoside Rb1 deglycosylation and compound K formation [33]. For a quantitative description of the main pharmacokinetic parameters, as well as gender-related differences, see Table 1.

Clinical studies investigating the pharmacokinetic profile of parenteral *P. notoginseng* are also available. In one of these studies, 12 healthy male volunteers were treated with *P. notoginseng* [as Xueshuantong, a formulation containing ginsenosides Rb1, Rd, Rg1, Re, and R1, obtained from Guangxi Wuzhou Pharmaceutical group (Wuzhou, Guangxi Zhuang Autonomous Region, China)] at the single dose of 150 mg either intramuscular (i.m.) or as 1.5 h intravenous (i.v.) infusion [34]. Unlike oral administration, parenteral *P. notoginseng* did not undergo first-pass metabolism in the gut, and unchanged ginsenosides were detected in the plasma, the most abundant being Rb1 [34]. This latter exhibited a mean C_max_ of 6.1 µM and 9 µM with T_max_ of 5 h and 1.5 h for i.m. and i.v. administration, respectively [34]. Ginsenoside Rb1 underwent slow hepatobiliary excretion, and only 32–38% was excreted unchanged into the urine with T_1/2_ of 46–57 h [34]. Similar results were described by Pintusophon et al., who treated 36 healthy volunteers (24 men and 12 women) with 250–500 mg Xueshuantong 2.5 h i.v. infusion [35]. Once again, Rb1 was the most abundant ginsenoside detected in plasma with mean C_max_ of 16–20 µM and 26–29 µM in subjects treated with 250 mg and 500 mg Xueshuantong, respectively [35]. About 40% of Rb1 was excreted in the urines with T_1/2_ of 40–43 h [35]. For a quantitative description of the main pharmacokinetic parameters, as well as gender-related differences, see Table 1. Unfortunately, no clinical studies describing the pharmacokinetics of steamed *P. notoginseng* are currently available.

Ginsenosides are metabolized in the liver, mainly through the 3A4 isoform of cytochrome P-450 (CYP), and excreted mainly through the feces, although the contribution of the kidney has been described (see above) [8,36].

## 4. Pharmacodynamics of *P. notoginseng*

Despite the extensive literature on the pharmacological effects of *P. notoginseng*, many studies provide only limited information because they report neither the exact chemical composition (e.g., ginsenoside type, amount) nor the degree of purification of the extract used. According to this premise, the current section focuses on the main preclinical studies describing the pleiotropic effects of *P. notoginseng*.

### 4.1. Immune System

The modulation of the immune response is one of the most beneficial effects of *P. notoginseng*. An ethanol extract from the *P. notoginseng* root, containing high levels of ginsenosides Rb1 and Rg1 (35% and 34% of the whole extract, respectively) at the doses 5–50 μg/mL, inhibited the lipopolysaccharide (LPS)-induced tumor necrosis factor (TNF)-α and interleukin (IL)-6 production in the murine dendritic cell line DC2.4 [37]. Interestingly, ginsenoside Rg1 (50 μg/mL) inhibited the LPS-stimulated cytokine production by DC2.4 cells more effectively than Rb1 [37].

An ethanol extract of *P. notoginseng*, rich in ginsenosides R1, Rg1, Re, Rb1, and Rd (total content 76.20%), at the concentration of 200 µg/mL, decreased oxidative stress markers [nitric oxide (NO) and ROS] and enhanced the cell stress response (total glutathione and xanthine oxidase) in RD4/2 cells infected with porcine circovirus 2 (PCV2) [38]. A similar decrease in ROS levels and enhancement of the cell stress response was detected in spleen lymphocytes of mice treated with 200 mg/kg intraperitoneal (i.p.) *P. notoginseng* extract 7 days after PCV2 infection [38].

*P. notoginseng* extract, in the range of 16–256 µg/mL, dose-dependently inhibited the replication of porcine reproductive and respiratory syndrome virus (PRRSV) in monkey Marc-145 cells [39]. Furthermore, at the dose of 10 mg/kg *per os*, this extract improved the immune response in piglets immunized with the anti-PRRSV JXA1-R vaccine, as observed by a marked decrease in PPRSV blood and tissue loads [39]. An ex vivo arm of this study confirmed that the *P. notoginseng* extract upregulated interferon (IFN)-α and IFN-β production in piglet peripheral blood mononuclear cells isolated before the challenge and exposed to PPRSV [39].

Andrographolide was used as an adjuvant to potentiate the immunostimulating activity of influenza vaccine [40]. As reported by Zhao et al., andrographolide (800 µg) boosted the immune response (e.g., splenocyte proliferation, bone marrow dendritic cell maturity, and both Th1 and Th2 cytokine secretion) in female Balb/c mice treated with the quadrivalent influenza vaccine [40].

An ethanol extract of steamed *P. notoginseng* (30–90 µM) inhibited migration and enhanced neutrophil apoptosis in a zebrafish tail-fin amputation model [24]. In particular, Rk3, Rh4, and Rg3 ginsenosides—formed following the steaming procedure, as mentioned above—were the major compounds involved in the neutropenic response [24].

### 4.2. Inflammation

Through the inhibition of cytokines, kinases, and transcription factors, *P. notoginseng* exerts significant anti-inflammatory effects. An *n*-buthanol extract of *P. notoginseng*, BT-201, containing ginsenosides Rb1 and R1 (24.1% and 8.4%, respectively), in the dose range 0.125–0.25 mg/mL, inhibited TNF-α, IL-1β, inducible nitric oxide synthase (iNOS)-derived NO, and matrix metalloproteinase (MMP)-13 release in both human monocytic THP-1 cells and mouse RAW264.7 macrophages [41]. Furthermore, BT-201 (0.25–2 mg/mL) counteracted the inflammatory response by inhibiting the activation of the inhibitor of nuclear factor κB (NF-κB) kinase subunit β (IκKβ), extracellular signal-regulated kinase (ERK), p38, and c-jun *N*-terminal kinase in THP-1 cells [41]. In a rheumatoid arthritis mouse model, such as collagen-induced arthritis, BT-201 (15 mg/kg/day *per os* for 18 days) delayed the onset of arthritis from 2.4 to 9.5 days and reduced both the edema and swelling of the paws [41].

In a streptozotocin-induced diabetic rat model, a *P. notoginseng* extract (containing ginsenosides R1, Rg1, Re, Rb1, and Rd) at the doses of 40, 80, 160 mg/kg *per os* for 2 months, counteracted the increased serum levels of TNF-α, IL-6, and IL-1β and inhibited the overexpression of phosphorylated IκKβ, IκB, and p65, in the ocular tissue [42]. Interestingly, ginsenosides Rg1 and Rb1 were detected in the ocular tissue at higher levels than the other saponins [42]. In an in vitro arm of this study, ginsenosides R1, Rg1, Re, Rb1, and Rd (25 µM) suppressed the inflammatory responses by blunting NF-κB activation and signaling pathways in MIO-M1 cells [42].

An extract of *P. notoginseng* root (major constituents ginsenosides Rb1 and Rd, 67.38% and 15.94%, respectively), at the doses of 50–200 mg/kg intragastric for 7 days, markedly improved clinical parameters (e.g., body weight loss, degree of diarrhea, and bloody feces) in a murine model of ulcerative colitis [43]. In dextran sulfate sodium-treated mice, *P. notoginseng* extract downregulated TNF-α, IL-1β, IL-6, and IL-18 mRNA expression and reversed the overexpression of inflammasome-related proteins, such as the NOD-, LRR-, and pyrin domain-containing protein 3 (NLRP3) and the apoptosis-associated speck-like protein containing a caspase-recruitment domain (ASC), as well as the cleaved caspase-1/procaspase-1 ratio in the colon tissue [43]. In addition, the *P. notoginseng* extract also suppressed the expression and translocation of high mobility group box 1 (HMGB1), thus interrupting the downstream toll-like receptor (TLR4)/NF-κB pathway [43]. Intriguingly, compound K (60 µM) exhibited significant anti-inflammatory effects and interfered with the TLR4-binding domain of HMGB1 in THP-1 and HT29 cells [43]. At the concentration of 30 µM, compound K inhibited the activation of the TLR4/NF-κB/NLRP3 inflammasome pathway in HMGB1-exposed THP-1 macrophages [43].

### 4.3. Cardiovascular System

In a preclinical model of blood deficiency syndrome (obtained by injecting 0.07 g/kg cyclophosphamide for 3 days and then 0.02 g/kg acetylphenylhydrazine on the fourth day), mice were treated with steamed *P. notoginseng* powder (0.45–1.8 mg/kg *per os* for 12 days) [44]. Steamed *P. notoginseng* supplementation increased erythropoietin and its receptor, thrombopoietin, granulocyte-macrophage colony-stimulating factor, and GATA-1 levels in mouse bone marrow, thus reversing anemia as shown by the normalization of leukocyte, erythrocyte, and platelet counts [44]. Furthermore, steamed *P. notoginseng* favored the cell cycle reaction and activation of immune cells through the JAK-STAT pathway in mouse spleen, which could promote hematopoiesis [44].

Together with the blood-tonifying effect, steamed *P. notoginseng* has significantly more potent antiplatelet and anticoagulant effects than the raw extract. In a collagen-induced platelet aggregation model, steamed *P. notoginseng* extract (3 mg/mL) inhibited platelet aggregation of 55–89% in rabbit blood samples with greater efficacy than a raw extract [45]. The antiplatelet effect increased with steaming duration and, after 9 h, was comparable to that produced by 200 µM acetylsalicylic acid (ASA) [45]. The efficacy of *P. notoginseng* in inhibiting platelet aggregation is higher than that of *P. ginseng* and *P. quinquefolius* [45]. Steamed *P. notoginseng* extract (3.3–10 mg/mL) increased prothrombin time (PT), activated partial thromboplastin time (APTT), and thrombin time more than the raw extract at each of the tested concentrations [45]. The antiplatelet and anticoagulant activity of steamed *P. notoginseng* extract, described in reconstituted systems, was confirmed in preclinical models. In Sprague-Dawley rats, a steamed *P. notoginseng* extract (500 mg/kg *per os*) inhibited platelet aggregation and increased bleeding time to a greater extent than the raw extract; interestingly, the percentage of platelet inhibition was not significantly different from the treatment with 25 mg/kg ASA [45]. The mechanisms involved in steamed *P. notoginseng* antiplatelet and anticoagulant effects are diverse and related to the ginsenoside composition (Table 2).

In a preclinical model of atherosclerosis, such as apolipoprotein E knockout (ApoE-KO) mice fed with high-fat diet, *P. notoginseng* freeze-drying powder (containing ginsenosides R1, Rb1, Rg1, Re, and Rd, total concentration 85.3%), at the dose of 60 mg/kg i.p. for 8 weeks, reduced plaque area and lipid deposition in the atheroschlerotic lesions in the aorta [50,51]. In addition, *P. notoginseng* reduced total cholesterol, low-density-lipoprotein cholesterol, and triglyceride blood levels in the treated animals, as well as the inflammatory response, as demonstrated by the inhibition of MMP-9, TIMP-1, and IL-1β [50,51]. In this animal model, the effect of *P. notoginseng* on atherosclerosis can be comparable to that of 20 mg/kg simvastatin [50]. An equivalent reduction in lipid deposition and inflammatory response was obtained with oral *P. notoginseng* powder [52].

A *P. notoginseng* extract (containing ginsenosides R1, Rg1, Re, Rb1, and Rd) at the dose of 100 mg/kg/day i.p. for 2 weeks improved post-myocardial infarction survival in mice with permanent ligation of left anterior descending coronary artery (70–77% versus 45.4% in untreated animals) [53]. Furthermore, *P. notoginseng* treatment significantly reduced the fibrotic scar size and increased the left ventricular wall thickness, thus preventing cardiac remodeling [53]. From a mechanistic viewpoint, *P. notoginseng* extract enhanced glucose deprivation-induced autophagy through the phosphorylation of 5’ adenosine monophosphate-activated protein kinase (AMPK)-Thr172 and calcium/calmodulin-stimulated protein kinase II-Thr287 in cardiomyocytes [53].

In rats with middle cerebral artery occlusion (MCAO), a well-known animal model of ischemic stroke, a *P. notoginseng* extract (containing ginsenosides R1, Rg1, Re, Rb1, and Rd) in the dose range 40–80 mg/kg intragastric, reduced lipid peroxidation and potentiated the antioxidant activity in the brain tissue [54]. In addition, *P. notoginseng*-treated rat brains exhibited marked upregulation of pro-survival pathways, including those related to either insulin growth factor 1 or phosphorylated Akt or phosphorylated mammalian target of rapamycin (mTOR) [54]. Furthermore, in the same animal model, a not better characterized methanol extract of *P. notoginseng*, at the dose of 50 mg/kg i.p. 2 h after MCAO, reduced brain infarct volume and counteracted inflammation, as shown by a marked reduction of iNOS, cyclooxygenase (COX)-2, and NF-κB [55].

### 4.4. Central Nervous System

Rats treated with *P. notoginseng* powder (2.5 g/kg/day) 3 days before and 3 days after traumatic brain injury (free-fall method) exhibited a significant improvement in tissue injury and neural function at 72 h paralleled by a marked increase in mTOR and phosphorylated mTOR [56]. This neuroprotective effect of *P. notoginseng* has been associated with a mTOR-dependent down-regulation of autophagy, as demonstrated by the reduction of sequestosome 1, beclin 1, and microtubule-associated protein 1 light chain 3 [56]. In the same experimental model, *P. notoginseng* reduced both the severity of hemorrhage and brain inflammation, as observed by the reduced levels of PT and APTT, together with the inhibition of brain NF-κB [57]. Saponins from the leaves of *P. notoginseng* (2.5–5–10 µg/mL), characterized by an increased content of ginsenoside Rc and Rb3, with a much lower content of ginsenosides Rb1, Rb2, and Rd, reduced 500 µM H_2_O_2_-induced cell death in primary cultures of rat cortical astrocytes [58]. The antioxidant effects of *P. notoginseng* in astrocytes were associated with the activation of nuclear factor erythroid 2-related factor 2 (Nrf2), a transcription factor that regulates the expression of several genes involved in the adaptive response to xenobiotics, including herbal products [58,59,60]. In particular, *P. notoginseng* activated the heat shock response, as demonstrated by the enhanced expression of heme oxygenase-1, a promising target for neuroprotective agents [58,61,62]. *P. notoginseng*-related neuroprotection was confirmed in the human neuroblastoma cell line SH-SY5Y exposed to oxygen and glucose deprivation/reoxygenation damage [58]. The neuroprotective effects of *P. notoginseng* should not only be ascribed to saponins because polyacetylene compounds also play an important role. As shown by Wang et al., panaxynol (8 µM) promoted neurite outgrowth in NGF-differentiated PC12D cells with a dual mechanism involving both protein kinase A and mitogen-activated protein kinase (MAPK) activation [63].

Beneficial effects of *P. notoginseng* have also been reported in preclinical models of neurodegenerative disorders. Through the inhibition of mTOR signaling and autophagy activation, *P. notoginseng* saponins (R1, Rg1, and Rb1), at the concentration of 0.1–1 µg/mL, exerted neuroprotective effects in PC12 cells exposed to 20 µM β-amyloid (Aβ), an in vitro model of Alzheimer’s disease (AD) [64]. Ginsenoside R1 (5–25 mg/kg/day by gavage for 3 months), the third most abundant saponin in *P. notoginseng*, increased choline acetyltransferase expression and improved cognitive function in the APP/PS1 double-transgenic mouse model of AD [65]. In this model, the mechanisms underlying the nootropic effect of ginsenoside R1 involve the upregulation of the insulin-degrading enzyme and the resulting inhibition of Aβ accumulation, both mediated by peroxisome proliferator-activated receptor-γ activation [65]. Ginsenoside Rg1 (10 mg/kg/day i.p. for 3 months) reduced brain Aβ due to a marked inhibition of γ-secretase activity and preserved spatial learning and memory in transgenic AD mice overexpressing amyloid precursor protein (Tg mAPP) [66]. In these animals, ginsenoside Rg1 enhanced the protein kinase A/cAMP response element-binding protein pathway activation, thus favoring synaptic function [66].

*P. notoginseng* saponins Rg1, R1, and Re (100 mg/kg twice daily i.p. for 7 days) ameliorated the locomotor deficit in male Kunming mice treated with the dopaminergic toxin 1-methyl-4-phenyl-1,2,3,6-tetrahydropyridine (MPTP), a widely used pharmacological tool to induce Parkinson’s disease (PD) [67]. In addition, these ginsenosides counteracted MPTP-induced neuronal death in the *substantia nigra pars compacta* through the overexpression of thioredoxin reductase-1, a well-known player in the cell stress response, and the down-regulation of inflammatory COX-2 [67,68]. In another preclinical model of PD, namely PC12 cells exposed to purified 1-methyl-4 phenylpyridinium (MPP^+^), a metabolite of MPTP, ginsenoside Rb1 protected PC12 cells from caspase-3-dependent apoptosis through stimulation of the estrogen receptor and the following activation of ERK1/2 and Akt and inhibition of stress-activated protein kinase/JNK and p38 pathways [69].

### 4.5. Metabolism

In a mouse model of type 2 diabetes mellitus, an ethanolic extract of *P. notoginseng* (rich in ginsenosides R1, Rb1, Re, Rg1, and Rd, 97.57%) at the dose of 20 mg/kg/day *per os* for 4 weeks, improved endothelium-dependent relaxations and alleviated endoplasmic reticulum stress and oxidative stress in aortas [70]. *P. notoginseng* was also effective in improving glucose sensitivity and normalizing blood pressure in diabetic mice [70]. The signaling pathways involved in these vasoprotective effects were those related to AMPK and endothelial NOS [70]. In rats treated with alloxan (40 mg/kg i.v.), a preclinical model of type 1 diabetes mellitus, *P. notoginseng* saponins (no characterization was provided), at the dose of 10–200 mg/kg/day intragastric for 3 months reduced glycemia and lipid levels and restored kidney function [71]. Furthermore, *P. notoginseng* treatment also restored the expression of both the bone morphogenetic protein-7 and plasminogen activator inhibitor-1, whose levels were reduced and increased, respectively, in the fibrotic kidney [71]. In rat mesangial cells, *P. notoginseng* saponins (100 µg/mL) confirmed the anti-fibrotic effect by suppressing the transcription of transforming growth factor-β1 and monocyte chemoattractant protein-1, reversing the increased acetylation of NF-κB p65 by high glucose and increasing sirtuin-1 and superoxide dismutase (SOD) expression [71].

In a rodent model of obesity, such as mice fed on a high-fat diet (60% fat, 20% protein, 20% carbohydrate) for 2 weeks, *P. notoginseng* saponins (R1, Rg1, and Rb1, 83.84%), at the doses of 400 or 800 mg/kg/day *per os* for 7 weeks, modified the gut microbiota by increasing the abundance of *Akkermansia muciniphila* and *Parabacteroides distasonis* [72]. Because of this reshaping, and due to the activation of the leptin-AMPK/STAT3 signaling pathway, an increase occurred in brown adipose tissue thermogenesis and beige adipocyte reconstruction [72]. Lastly, reduced host adiposity and promoted energy expenditure were observed in these animals [72].

### 4.6. Cancer

*P. notoginseng* saponins Rb1, Rg1, Rd, R1, and Rh1, in the dose range 50–400 μg/mL, reduced the growth of Lewis-lung carcinoma (LLC) cell line, and mitigated the development of tumors in LLC cell-inoculated mice, by down-regulating a wide array of genes involved in malignant transformation, including Braf1, Cdk6, Notch3, Met, Col1a1, Hgf, Raf1, and Scd1 [73].

An ethanol extract of P. notoginseng, rich in ginsenoside R1 and Rg2, in the dose range 1–5 mg/mL, arrested the cell cycle at the G2/M phase in both prostate cancer LNCaP and 22Rv1 cell lines [74]. In addition, *P. notoginseng*-treated PCa cells released less tumor-promoting IL-4 than controls [74].

Intriguingly, steamed *P. notoginseng* (0.25 mg/mL) displays greater antiproliferative effects on liver cancer SNU449, SNU182, and HepG2 cells than the raw form [75].

Panaxynol and panaxydol (2 and 5 µM) have been shown to inhibit the proliferation of HL60 cells, a human promyeloblast cell line, through the activation of caspase-3 and the following cleavage of poly(ADP-ribose) polymerase [76].

With regard to colorectal cancer (CRC), *P. notoginseng* whole extract and ginsenoside R1 have been shown to inhibit migration, invasion, and adhesion of the human cell line HCT-116 [77,78]. The anti-metastatic properties of ginsenoside R1 (75–300 µM) rely on the inhibitory effect on adhesion molecules such as MMP-9, integrin-1, E-selectin, and intercellular adhesion molecule-1 [77,78]. *P. notoginseng* exerts adjuvant effects in CRC, as demonstrated by the evidence that *n*-butanol extract rich in ginsenosides Rb1, Rb2, Rb3, and Rc at the dose of 0.25 mg/mL enhances the antiproliferative effect of 5-fluorouracil on HCT-116 cancer cells and may decrease the dosage of the antiblastic drug [79].

Similar anti-metastatic properties of *P. notoginseng* saponins have been confirmed in an in vivo model of breast cancer. As shown by Wang et al., a mixture of ginsenosides Rb1, Rg1, Rd, R1, and Rh1 (50–400 µg/mL) induces apoptosis and cell cycle arrest of highly-metastatic mouse breast cancer 4T1 cells [80]. In this cell line, *P. notoginseng* increases the expression of proteins that suppress tumor metastasis, including breast cancer metastasis suppressor 1, metastasis suppressor 1, and tissue inhibitor of metalloproteinase 2 [80]. Comparable results have been shown in Balb/c mice injected with 4T1 cells, treated with *P. notoginseng* saponins (150 mg/kg i.p.), and sacrificed 3 weeks later [80].

## 5. Clinical Studies

Sanchitongshu is a Chinese medicine extracted from *P. notoginseng* and considerably rich in PPT-derived ginsenosides (~80%), 60% of which is Rg1 and the remainder Re (6%) and R1 (11%) [81]. In a multi-center, double-blind, randomized clinical trial (RCT), 140 patients with ischemic stroke in anterior cerebral circulation were allocated to receive either ASA (50 mg daily) and 200 mg Sanchitongshu capsules (100 mg saponins and 100 mg inactive starch, Pharmaceutical Factory of Chengdu Hoist Inc. Ltd., Chengdu, China) three times daily, or ASA as above and placebo capsules for 4 weeks [81]. Patients treated with the combination of ASA and Sanchitongshu achieved a significant improvement in both neurological deficits and activities of daily living compared with patients receiving ASA and placebo [81]. Neither severe adverse effects nor deaths were reported during the RCT [81]. Only eight patients in the Sanchitongshu arm and six in the placebo arm reported nausea, gastric discomfort, and increased frequency of evacuation lasting 2–7 days [81].

Xuesaitong, another Chinese medicine formula from *P. notoginseng* containing saponins Rg1 (35,3%), Rb1 (32.3%), R1 (9.80%), Rd (4.9%), Re (4%), and the remainder being other ginsenosides, has proven effective in ischemic stroke, cerebral infarction, and unstable angina pectoris [82,83]. In a recent multi-center, double-blind RCT, 2966 patients with ischemic stroke were assigned to receive either Xuesaitong soft capsules (120 mg twice daily, manufactured by China Resources Kunming Shenghuo Pharmaceutical Co., Ltd., Kunming, China) or placebo for 3 months [84]. Patients treated with *P. notoginseng* exhibited a significant improvement in neurological deficits and better functional independence than those receiving placebo (89.3% vs. 82.4%, respectively) [84]. With regard to the safety profile, serious adverse events arose in 1.0% of patients receiving *P. notoginseng* and 1.1% in the control group [84]. Xuesaitong has also shown great effectiveness in patients with cerebral infarction. A recent meta-analysis of 7 RCTs involving 827 patients concluded that Xuesaitong, as an add-on to conventional treatments (antihypertensive, hypoglycemic, hypolipidemic, antithrombotic, etc.), improved both clinical outcomes, such as the neurological deficit score and number of cured case, and blood rheology indicators, including whole blood viscosity, fibrinogen, and hematocrit, in patients with acute cerebral infarction [82]. In the analyzed studies, no significant adverse reactions were reported. Likewise, a meta-analysis of 17 RCTs involving 2315 patients revealed that Xuesaitong combined with conventional medicines [ASA, β-blockers, angiotensin-converting enzyme inhibitors, angiotensin receptor blockers, statins, calcium-channel blockers, nitrates, etc.] significantly reduced mortality in patients with unstable angina with respect to those receiving only conventional treatment [85]. In addition, Xuesaitong significantly reduced the duration of angina attacks and the dosage of nitroglycerin and improved both the electrocardiographic abnormalities and plasma lipid profile compared to conventional treatment [85]. No adverse events related to Xuesaitong were reported in this study [85]. In a single center, assessor-blinded RCT, 89 patients with stable coronary heart disease (SCHD) and endoscopy-confirmed chronic gastritis were assigned 100 mg of ASA daily or 60 mg of Xuesaitong capsules [Kunming Shenghuo Pharmacy Co., Ltd. (Kunming, China)] twice daily in addition to 100 mg of ASA for 8 weeks [86]. Compared with ASA alone, Xuesaitong + ASA suppressed platelet COX-1 activity and decreased TXA_2_ production [86]. Furthermore, patients in the Xuesaitong arm exhibited a marked relief in dyspepsia-related symptoms, probably by increasing both gastrin and motilin release [86]. No significant adverse effects were reported, with the exception of one patient in the Xuesaitong group, who experienced mild and transient constipation [86].

## 6. Interactions and Precautions

As mentioned in Section 3, *P. notoginseng* saponins are mainly metabolized through CYP3A4 [36], the isoform responsible for the metabolism of most drugs, and, therefore, several studies were carried out to explore the potential interaction between ginsenosides and this isozyme. Most available data derived from ex vivo studies show that *P. notoginseng* does not have any significant effect on the metabolism of conventional drugs metabolized through CYP3A4 [87,88]. Similarly, negligible interactions have been reported in terms of induction/inhibition of CYP2C9, CYP2C19, CYP2B6, and CYP2E1 by *P. notoginseng* [87,89]. Only limited information is available on the modulation of either phase II drug-metabolizing enzymes or transporters by *P. notoginseng*. Ginsenoside Rg3 inhibited UDP-glucuronosyltransferase isoforms 1A7, 1A8, 2B7, and 2B15 in a reconstituted system (K_i_ 22.6, 7.9, 1.9, and 2.0 μM, respectively), whereas saponins Rh2, Rg3, and R1 inhibited P-glycoprotein (P-gp), thus reversing resistance to many drugs [87,90]. In particular, ginsenoside Rh2 (5–50 mg/kg intragastric or 10 µM) increased the oral absorption of digoxin in rats and increased the bioavailability of ritonavir in Caco-2 and MDCK-MDR1 cells [87,91]. The last ginsenoside, through either the inhibition of P-gp and NorA efflux pumps or the stabilization of the gyrase-fluoroquinolone complex, fosters the antibacterial effects of ciprofloxacin and levofloxacin towards susceptible bacteria, such as *S. aureus* [92,93,94]. Lastly, a *P. notoginseng* suspension [containing ginsenosides R1 (6.9%), Rg1 (28.0%), Rb1 (29.7%), Re (3.8%), and Rd (7.3%)], at the dose of 30.25 mg/kg *per os*, increased the systemic bioavailability of ASA (20.83 mg/kg *per os*) in Sprague-Dawley rats [95]. The mechanism for such an interaction relies on the ability of saponins to increase ASA transport across the cell membrane [95]. Although not yet proven in humans, this mechanism may increase the risk of bleeding in subjects treated with low-dose ASA who, at the same time, take *P. notoginseng* as a dietary supplement.

From a pharmacodynamic viewpoint, interactions of *P. notoginseng* with antiblastic drugs have been described. Ginsenoside Rb1 (250 nM) sensitized cancer stem/tumor-initiating cells to pharmacological doses of cisplatin and paclitaxel through the down-regulation of β-catenin/T-cell factor-dependent transcription and the expression of its target genes ABCG2 and P-gp [87,96]. In the A549 lung cancer cell line, ginsenoside Rd (80 µM) attenuated 20 μM cisplatin resistance and significantly increased the inhibitory rate of cell invasion and proliferation through an Nrf2-dependent mechanism [87,97]. In triple-negative MDA-MB-231 breast cancer cell lines, 10 µM ginsenoside Rg1 enhanced doxorubicin (DOX)-dependent apoptosis through a mechanism involving MAPK, Bax, caspase-3, and caspase-9 overexpression [87,98]. Similarly, ginsenoside Rg1 (80 mg/kg/day *per os* 1 week before and then until the sacrifice) mitigated DOX (15 mg/kg i.p.)-related cardiomyopathy by increasing Akt and ERK phosphorylation and Bcl-2/Bax ratio in mice [87,99].

Although these results support the idea that *P. notoginseng* could enhance both the pharmacological or toxic effects of conventional drugs, these lines of evidence mainly derive from preclinical studies. The lack of significant data collected through clinical studies does not allow us to draw any conclusion on the potential herb–drug interaction of *P. notoginseng*.

## 7. Toxicology

The acute toxicity of raw and decocted extracts of *P. notoginseng* has been studied in zebrafish *larvae*. Both the raw and decocted *P. notoginseng* extracts dose-dependently killed larval zebrafish, with an estimated lethal dose (LD)_50_ of 73.8 µg/mL and 151 µg/mL, respectively [100]. Significant abnormalities in body weight, body length, and number of *vertebrae* were detected in *larvae* after 21 days of treatment with the decocted extract [100]. However, the developmental toxicity of the decocted *P. notoginseng* extract seems to be related to neither the main compounds ginsenoside R1 nor the non-protein amino acid L-dencichine [100]. Taking into consideration the lack of studies carried out in rodents, the main species for the study of xenobiotic acute toxicity, and the limited evidence provided by the study described above, it is not possible to draw any relevant conclusion on *P. notoginseng* acute toxicity.

With regard to sub-chronic toxicity, *P. notoginseng* powder (0.75–2.37–7.5 g/kg/day) was administered by gastric gavage for 90 days to 40 male Sprague-Dawley rats [101]. Neither physical nor behavioral abnormalities were observed in treated animals [101]. Compared to control rats, no significant differences were reported in body weight, total and differential white blood cell counts, total and albumin serum protein levels, transaminases, natural killer cell activity, lymphocyte transformation, spleen and thymus weight [101].

As far as the reproductive and genetic toxicity of *P. notoginseng* is concerned, unfortunately, no specific studies are available. The only evidence found in the literature is a study by Zhang et al., who evaluated the effects of *P. notoginseng* on platinum-based reproductive and genetic toxicity [102]. The Authors reported that a *P. notoginseng* preparation containing ginsenosides Rg1, Rb1, and R1, administered at the doses 0.19–0.58 g/kg/day for 14 days by gavage, counteracted cisplatinum-induced reduction in body weight and testis and epididymis weight in male Kunming mice [102]. In addition, *P. notoginseng* increased sperm count/viability and decreased abnormal sperm morphology in cisplatin-treated animals [102]. Lastly, *P. notoginseng* reduced both the tail moment and micronucleus formation in peripheral blood cells and bone marrow cells, respectively, in cisplatin-treated mice [102].

## 8. Conclusions and Future Directions

As reported in the pharmacodynamic studies summarized above, *P. notoginseng* exerts beneficial effects by acting at several levels. The beneficial effects described on immune-inflammatory, cardiovascular, neurodegenerative and metabolic diseases, as well as in cancer, make *P. notoginseng* an important herbal product. Unfortunately, despite the abundance of preclinical data, few clinical trials supported the therapeutic role of *P. notoginseng*, mainly as an add-on therapy for the treatment of cardiovascular diseases. The good news from these studies is that, at the controlled doses, *P. notoginseng* has nearly no toxic effects. Some concerns arise when considering the folk use of *P. notoginseng* as a dietary supplement. In 1979, Siegel reported a “ginseng abuse syndrome” characterized by CNS hyperactivity, skin eruptions, and morning diarrhea in patients taking high-dose ginseng (up to 15 g/day) [103]. These lines of evidence, together with the limited data about the toxicological profile of *P. notoginseng*, downsize its translational potential. Indeed, in 2020, the European Food Safety Agency (EFSA) considered a botanical extract derived from *P. notoginseng* and *Astragalus membranaceous* safe at the dose of 0.5 mg/kg/day in adults, excluding pregnant women [104]. Although promising, this EFSA report is based on data related to a mixed extract of both *P. notoginseng* and *Astragalus membranaceous*, without any possibility of identifying the profile of single products [104].

Sub-chronic and chronic studies carried out in male and female rodents and beagle dogs would help assess the effects of long-term exposure to *P. notoginseng*. In addition, these studies would allow us to calculate the lowest observed adverse effect level (LOAEL) and no observed adverse effect level (NOAEL), parameters necessary for deciding the dose to use in controlled human studies. Only thanks to this approach can ginseng arise from the niche of a dietary supplement or traditional Chinese medicine to gain drug status as suggested by preclinical studies.

## Figures and Tables

**Table 1:** Quantitative description of the main pharmacokinetic parameters of ginsenoside Rb1 and compound K in humans treated with different preparations of *P. notoginseng*. For further information, see Section 3.

Sources	C_max_ (µM)	T_max_ (h)	AUC_0-t_ (µM∙h)	T_1/2_ (h)	Reference
**Ginsenoside Rb1**					
Sanqui extract PO ^a^					
Female	0.021 ± 0.009	7.8 ± 3.7	0.67 ± 0.30	33–57	[32]
Male	0.035 ± 0.038	11.8 ± 2.9	0.83 ± 0.42		
Xueshuantong ^b^					
Intramuscular	6.06 ± 0.65	5.3 ± 2.4	254.42 ± 26.65	46.3 ± 9.6	[34]
Intravenous ^c^	8.83 ± 1.38	1.5	227.25 ± 16.12	57.0 ± 14.5	
Xueshuantong ^d^					
Female 250 mg	19.5 ± 2.2	2.5	492.4 ± 46.7	39.9 ± 4.7	[35]
Male 250 mg	16.3 ± 3.7		425.5 ± 68.0	43.5 ± 6.5	
Female 500 mg	28.9 ± 3.2		887.4 ± 97.5	41.0 ± 7.6	
Male 500 mg	26.3 ± 2.6		737.7 ± 99.3	42.9 ± 7.5	
**Compound K**					
Sanqui extract PO ^a^					
Female	0.324 ± 0.15	17.3 ± 6.4	5.71 ± 2.39	NC	[32]
Male	0.249 ± 0.18	10.9 ± 8.7	3.79 ± 2.91		

Data are expressed as mean ± standard deviation; ^a^ 270 mL; ^b^ 150 mg; ^c^ 1.5 h intravenous infusion; ^d^ 2.5 h intravenous infusion; AUC_0-t_, area under the curve_0-t_; C_max_, peak plasma concentration; N.C., not calculated. PO, *per os*; T_1/2_, half-life; T_max_, time to reach the C_max._

**Table 2:** Main mechanisms through which steamed *P. notoginseng* ginsenosides exert antiplatelet and anticoagulant effects. For further information, see Section 4.3.

Ginsenoside	Mechanism(s)	Reference(s)
Rk3	Inactivation of the integrin αIIb/β_3_ and reduction of fibrinogen binding. Inhibition of MAPK and PI3K/Akt phosphorylation leads to reduced production of TXA_2_.	[46]
Rg3	Increased cAMP levels. Suppressed thrombin-induced elevation of intracellular calcium levels. Inhibition of factor Xa.	[47,48]
Rg1	Inhibition of thrombin-induced ERK phosphorylation. Decreased rate of clot retraction.	[49]

cAMP, cyclic adenosine monophosphate; ERK, extracellular signal-regulated kinase; MAPK, mitogen-activated protein kinase; PI3K, phosphatidylinositol-3 kinase; TXA_2_, thromboxane A_2_.

## References

[1] Dennehy C.E., Tsourounis C., Katzung B.G. (2001). Botanicals (“Herbal medications”) & nutritional supplements. Basic & Clinical Pharmacology.

[2] Tayeb B.A., Kusuma I.Y., Osman A.A.M., Minorics R. (2024). Herbal compounds as promising therapeutic agents in precision medicine strategies for cancer: A systematic review. J. Integr. Med..

[3] de Waure C., Bertola C., Baccarini G., Chiavarini M., Mancuso C. (2023). Exploring the contribution of curcumin to cancer therapy: A systematic review of randomized controlled trials. Pharmaceutics.

[4] Grujić-Milanović J., Rajković J., Milanović S., Jaćević V., Miloradović Z., Nežić L., Novaković R. (2023). Natural substances vs. approved drugs in the treatment of main cardiovascular disorders-Is there a breakthrough?. Antioxidants.

[5] Islam A., Mishra A., Ahsan R., Fareha S. (2023). Phytopharmaceuticals and herbal approaches to target neurodegenerative disorders. Drug Res..

[6] Rahman M.M., Islam M.R., Shohag S., Hossain M.E., Rahaman M.S., Islam F., Ahmed M., Mitra S., Khandaker M.U., Idris A.M. (2022). The multifunctional role of herbal products in the management of diabetes and obesity: A comprehensive review. Molecules.

[7] Mhillaj E., Cuomo V., Trabace L., Mancuso C. (2019). The heme oxygenase/biliverdin reductase system as effector of the neuroprotective outcomes of herb-based nutritional supplements. Front. Pharmacol..

[8] Mancuso C., Santangelo R. (2017). Panax ginseng and Panax quinquefolius: From pharmacology to toxicology. Food. Chem. Toxicol..

[9] Mancuso C., Santangelo R. (2014). Ferulic acid: Pharmacological and toxicological aspects. Food. Chem. Toxicol..

[10] Mancuso C., Siciliano R., Barone E., Preziosi P. (2012). Natural substances and Alzheimer’s disease: From preclinical studies to evidence based medicine. Biochim. Biophys. Acta..

[11] Botanicals. https://www.efsa.europa.eu/en/topics/topic/botanicals.

[12] Botanical Medicine. https://www.takingcharge.csh.umn.edu/10-top-best-selling-botanicals-what-they-do.

[13] Baeg I.H., So S.H. (2013). The world ginseng market and the ginseng (Korea). J. Ginseng Res..

[14] Yun T.K. (2001). Brief introduction of Panax ginseng C.A. Meyer. J. Korean Med. Sci..

[15] Xu C., Wang W., Wang B., Zhang T., Cui X., Pu Y., Li N. (2019). Analytical methods and biological activities of Panax notoginseng saponins: Recent trends. J. Ethnopharmacol..

[16] Global Notoginseng Suppliers. https://www.thetradevision.com/global/notoginseng-suppliers.

[17] Global Notoginseng Root Extract Market Report. https://www.marketresearchintellect.com/product/global-notoginseng-root-extract-market-size-and-forecast.

[18] Liu H., Lu X., Hu Y., Fan X. (2020). Chemical constituents of Panax ginseng and Panax notoginseng explain why they differ in therapeutic efficacy. Pharmacol. Res..

[19] Chen W., Dang Y., Zhu C. (2010). Simultaneous determination of three major bioactive saponins of Panax notoginseng using liquid chromatography-tandem mass spectrometry and a pharmacokinetic study. Chin. Med..

[20] Wang T., Guo R., Zhou G., Zhou X., Kou Z., Sui F., Li C., Tang L., Wang Z. (2016). Traditional uses, botany, phytochemistry, pharmacology and toxicology of Panax notoginseng (Burk.) F.H. Chen: A review. J. Ethnopharmacol..

[21] Yang W.Z., Hu Y., Wu W.Y., Ye M., Guo D.A. (2014). Saponins in the genus Panax L. (Araliaceae): A systematic review of their chemical diversity. Phytochemistry.

[22] Kim D.H. (2012). Chemical Diversity of Panax ginseng, Panax quinquifolium, and Panax notoginseng. J. Ginseng Res..

[23] Liu J., Wang Y., Qiu L., Yu Y., Wang C. (2014). Saponins of Panax notoginseng: Chemistry, cellular targets and therapeutic opportunities in cardiovascular diseases. Expert. Opin. Investig. Drugs..

[24] Xiong Y., Halima M., Che X., Zhang Y., Schaaf M.J.M., Li M., Gao M., Guo L., Huang Y., Cui X. (2022). Steamed *Panax notoginseng* and its saponins inhibit the migration and induce the apoptosis of neutrophils in a zebrafish tail-fin amputation model. Front. Pharmacol..

[25] Sun S., Qi L.W., Du G.J., Mehendale S.R., Wang C.Z., Yuan C.S. (2011). Red notoginseng: Higher ginsenoside content and stronger anticancer potential than Asian and American ginseng. Food. Chem..

[26] Xiong Y., Chen L., Man J., Hu Y., Cui X. (2019). Chemical and bioactive comparison of *Panax notoginseng* root and rhizome in raw and steamed forms. J. Ginseng Res..

[27] Liu C., Zuo Z., Xu F., Wang Y. (2023). Study of the suitable climate factors and geographical origins traceability of *Panax notoginseng* based on correlation analysis and spectral images combined with machine learning. Front. Plant. Sci..

[28] Xu Y., Zhu M., Feng Y., Xu H. (2023). Panax notoginseng-microbiota interactions: From plant cultivation to medicinal application. Phytomedicine.

[29] Dennehy C.E., Tsourounis C.T., Katzung B.G., Vanderah T.W. (2021). Dietary supplements & herbal medications. Basic & Clinical Pharmacology.

[30] Lee J., Lee E., Kim D., Lee J., Yoo J., Koh B. (2009). Studies on absorption, distribution and metabolism of ginseng in humans after oral administration. J. Ethnopharmacol..

[31] Wang H.Y., Qi L.W., Wang C.Z., Li P. (2011). Bioactivity enhancement of herbal supplements by intestinal microbiota focusing on ginsenosides. Am. J. Chin. Med..

[32] Hu Z., Yang J., Cheng C., Huang Y., Du F., Wang F., Niu W., Xu F., Jiang R., Gao X. (2013). Combinatorial metabolism notably affects human systemic exposure to ginsenosides from orally administered extract of Panax notoginseng roots (Sanqi). Drug. Metab. Dispos..

[33] Wang S., Song F., Gu H., Shu Z., Wei X., Zhang K., Zhou Y., Jiang L., Wang Z., Li J. (2022). Assess the diversity of gut microbiota among healthy adults for forensic application. Microb. Cell. Fact..

[34] Zhang H.Y., Niu W., Olaleye O.E., Du F.F., Wang F.Q., Huang Y.H., Yuan L., Li Y.F., Liu G.P., Xu F. (2020). Comparison of intramuscular and intravenous pharmacokinetics of ginsenosides in humans after dosing XueShuanTong, a lyophilized extract of Panax notoginseng roots. J. Ethnopharmacol..

[35] Pintusophon S., Niu W., Duan X.N., Olaleye O.E., Huang Y.H., Wang F.Q., Li Y.F., Yang J.L., Li C. (2019). Intravenous formulation of Panax notoginseng root extract: Human pharmacokinetics of ginsenosides and potential for perpetrating drug interactions. Acta. Pharmacol. Sin..

[36] Qi L.W., Wang C.Z., Du G.J., Zhang Z.Y., Calway T., Yuan C.S. (2011). Metabolism of ginseng and its interactions with drugs. Curr. Drug. Metab..

[37] Rhule A., Rase B., Smith J.R., Shepherd D.M. (2008). Toll-like receptor ligand-induced activation of murine DC2.4 cells is attenuated by Panax notoginseng. J. Ethnopharmacol..

[38] Wang Q.H., Kuang N., Hu W.Y., Yin D., Wei Y.Y., Hu T.J. (2020). The effect of *Panax notoginseng* saponins on oxidative stress induced by PCV2 infection in immune cells: In vitro and in vivo studies. J. Vet. Sci..

[39] Yi H., Yu Z., Wang Q., Sun Y., Peng J., Cai Y., Ma J., Chen Y., Qin C., Cai M. (2022). Panax notoginseng saponins suppress type 2 porcine reproductive and respiratory syndrome virus replication in vitro and enhance the immune effect of the live vaccine JXA1-R in piglets. Front. Vet. Sci..

[40] Zhao D., Chen X., Wang L., Zhang J., Lv R., Tan L., Chen Y., Tao R., Li X., Chen Y. (2023). Improvement influenza vaccine immune responses with traditional Chinese medicine and its active ingredients. Front. Microbiol..

[41] Chang S.H., Choi Y., Park J.A., Jung D.S., Shin J., Yang J.H., Ko S.Y., Kim S.W., Kim J.K. (2007). Anti-inflammatory effects of BT-201, an n-butanol extract of Panax notoginseng, observed in vitro and in a collagen-induced arthritis model. Clin. Nutr..

[42] Wang Y., Sun X., Xie Y., Du A., Chen M., Lai S., Wei X., Ji L., Wang C. (2024). Panax notoginseng saponins alleviate diabetic retinopathy by inhibiting retinal inflammation: Association with the NF-κB signaling pathway. J. Ethnopharmacol..

[43] Chen J., Lu P., Liu J., Yang L., Li Y., Chen Y., Wang Y., Wan J., Zhao Y. (2023). 20(S)- Protopanaxadiol saponins isolated from Panax notoginseng target the binding of HMGB1 to TLR4 against inflammation in experimental ulcerative colitis. Phytother. Res..

[44] Zhang Z., Zhang Y., Gao M., Cui X., Yang Y., van Duijn B., Wang M., Hu Y., Wang C., Xiong Y. (2020). Steamed *Panax notoginseng* attenuates anemia in mice with blood deficiency syndrome via regulating hematopoietic factors and JAK-STAT pathway. Front. Pharmacol..

[45] Lau A.J., Toh D.F., Chua T.K., Pang Y.K., Woo S.O., Koh H.L. (2009). Antiplatelet and anticoagulant effects of Panax notoginseng: Comparison of raw and steamed Panax notoginseng with Panax ginseng and Panax quinquefolium. J. Ethnopharmacol..

[46] Kwon H.W., Shin J.H., Rhee M.H., Park C.E., Lee D.H. (2023). Anti-thrombotic effects of ginsenoside Rk3 by regulating cAMP and PI3K/MAPK pathway on human platelets. J. Ginseng Res..

[47] Kwon H.W. (2018). Inhibitory effect of 20(S)-Ginsenoside Rg3 on human platelet aggregation and intracellular Ca^2+^ levels via cyclic adenosine monophosphate dependent manner. Prev. Nutr. Food. Sci..

[48] Xiong L., Qi Z., Zheng B., Li Z., Wang F., Liu J., Li P. (2017). Inhibitory effect of triterpenoids from Panax ginseng on coagulation factor X. Molecules.

[49] Zhou Q., Jiang L., Xu C., Luo D., Zeng C., Liu P., Yue M., Liu Y., Hu X., Hu H. (2014). Ginsenoside Rg1 inhibits platelet activation and arterial thrombosis. Thromb. Res..

[50] Long L., Yu Z., Qu H., Wang N., Guo M., Zhou X., Fu C., Gao Z. (2020). Prediction of the network pharmacology-based mechanism for attenuation of atherosclerosis in apolipoprotein E knockout mice by *Panax notoginseng* saponins. Evid. Based Complement. Alternat. Med..

[51] Liu Y., Hao F., Zhang H., Cao D., Lu X., Li X. (2013). Panax notoginseng saponins promote endothelial progenitor cell mobilization and attenuate atherosclerotic lesions in apolipoprotein E knockout mice. Cell. Physiol. Biochem..

[52] Yang H., Liu Z., Hu X., Liu X., Gui L., Cai Z., Dai C. (2022). Protective effect of Panax Notoginseng saponins on apolipoprotein-E-deficient atherosclerosis-prone mice. Curr. Pharm. Des..

[53] Wang D., Lv L., Xu Y., Jiang K., Chen F., Qian J., Chen M., Liu G., Xiang Y. (2021). Cardioprotection of Panax Notoginseng saponins against acute myocardial infarction and heart failure through inducing autophagy. Biomed. Pharmacother..

[54] Wang C., Chen H., Ma S.T., Mao B.B., Chen Y., Xu H.N., Yu H. (2021). A network pharmacology approach for exploring the mechanisms of *Panax notoginseng* saponins in ischaemic stroke. Evid. Based Complement. Alternat. Med..

[55] Son H.Y., Han H.S., Jung H.W., Park Y.K. (2009). Panax notoginseng attenuates the infarct volume in rat ischemic brain and the inflammatory response of microglia. J. Pharmacol. Sci..

[56] Shi Y., Zhou X., Yang R., Ying S., Wang L. (2021). *Panax notoginseng* protects the rat brain function from traumatic brain injury by inhibiting autophagy via mammalian targeting of rapamycin. Aging.

[57] Jiang T., Zhou X., Jiang H., Ying R., Zhang Z., Cai D., Wu Y., Fang H., Wang L. (2021). Efficacy of Sanqi (Radix Notoginseng) in treating cerebral hemorrhage in rats with traumatic brain injury. J. Tradit. Chin. Med..

[58] Zhou N., Tang Y., Keep R.F., Ma X., Xiang J. (2014). Antioxidative effects of Panax notoginseng saponins in brain cells. Phytomedicine.

[59] Catino S., Paciello F., Miceli F., Rolesi R., Troiani D., Calabrese V., Santangelo R., Mancuso C. (2016). Ferulic Acid Regulates the Nrf2/Heme Oxygenase-1 System and Counteracts Trimethyltin-Induced Neuronal Damage in the Human Neuroblastoma Cell Line SH-SY5Y. Front. Pharmacol..

[60] Fetoni A.R., Paciello F., Rolesi R., Eramo S.L., Mancuso C., Troiani D., Paludetti G. (2015). Rosmarinic acid up-regulates the noise-activated Nrf2/HO-1 pathway and protects against noise-induced injury in rat cochlea. Free Radic. Biol. Med..

[61] Calabrese V., Mancuso C., Ravagna A., Perluigi M., Cini C., De Marco C., Butterfield D.A., Stella A.M. (2007). In vivo induction of heat shock proteins in the substantia nigra following L-DOPA administration is associated with increased activity of mitochondrial complex I and nitrosative stress in rats: Regulation by glutathione redox state. J. Neurochem..

[62] Mancuso C. (2022). The brain heme oxygenase/biliverdin reductase system as a target in drug research and development. Expert. Opin. Ther. Targets..

[63] Wang Z.J., Nie B.M., Chen H.Z., Lu Y. (2006). Panaxynol induces neurite outgrowth in PC12D cells via cAMP- and MAP kinase-dependent mechanisms. Chem. Biol. Interact..

[64] Jiang Y., Li S., Xie X., Li H., Huang P., Li B., Huo L., Zhong J., Li Y., Xia X. (2021). Exploring the mechanism of *Panax notoginseng* saponins against Alzheimer’s disease by network pharmacology and experimental validation. Evid. Based Complement. Alternat. Med..

[65] Li Z., Li H., Zhao C., Lv C., Zhong C., Xin W., Zhang W. (2015). Protective effect of notoginsenoside R1 on an APP/PS1 mouse model of Alzheimer’s disease by up-regulating insulin degrading enzyme and inhibiting Aβ accumulation. CNS. Neurol. Disord. Drug. Targets..

[66] Fang F., Chen X., Huang T., Lue L.F., Luddy J.S., Yan S.S. (2012). Multi-faced neuroprotective effects of Ginsenoside Rg1 in an Alzheimer mouse model. Biochim. Biophys. Acta.

[67] Luo F.C., Wang S.D., Qi L., Song J.Y., Lv T., Bai J. (2011). Protective effect of panaxatriol saponins extracted from Panax notoginseng against MPTP-induced neurotoxicity in vivo. J. Ethnopharmacol..

[68] Calabrese V., Cornelius C., Mancuso C., Pennisi G., Calafato S., Bellia F., Bates T.E., Giuffrida Stella A.M., Schapira T., Dinkova Kostova A.T. (2008). Cellular stress response: A novel target for chemoprevention and nutritional neuroprotection in aging, neurodegenerative disorders and longevity. Neurochem. Res..

[69] Hashimoto R., Yu J., Koizumi H., Ouchi Y., Okabe T. (2012). Ginsenoside Rb1 prevents MPP(+)-induced apoptosis in PC12 cells by stimulating estrogen receptors with consequent activation of ERK1/2, Akt and inhibition of SAPK/JNK, p38 MAPK. Evid. Based Complement. Alternat. Med..

[70] Zhang X., Zhou C., Miao L., Tan Y., Zhou Y., Cheong M.S., Huang Y., Wang Y., Yu H., Cheang W.S. (2021). *Panax notoginseng* protects against diabetes-associated endothelial dysfunction: Comparison between ethanolic extract and total saponin. Oxid. Med. Cell. Longev..

[71] Du Y.G., Wang L.P., Qian J.W., Zhang K.N., Chai K.F. (2016). Panax notoginseng saponins protect kidney from diabetes by up-regulating silent information regulator 1 and activating antioxidant proteins in rats. Chin. J. Integr. Med..

[72] Xu Y., Wang N., Tan H.Y., Li S., Zhang C., Zhang Z., Feng Y. (2020). *Panax notoginseng* saponins modulate the gut microbiota to promote thermogenesis and beige adipocyte reconstruction via leptin-mediated AMPKα/STAT3 signaling in diet-induced obesity. Theranostics.

[73] Yang Q., Wang P., Cui J., Wang W., Chen Y., Zhang T. (2016). Panax notoginseng saponins attenuate lung cancer growth in part through modulating the level of Met/miR-222 axis. J. Ethnopharmacol..

[74] Hawthorne B., Lund K., Freggiaro S., Kaga R., Meng J. (2022). The mechanism of the cytotoxic effect of Panax notoginseng extracts on prostate cancer cells. Biomed. Pharmacother..

[75] Toh D.F., Patel D.N., Chan E.C., Teo A., Neo S.Y., Koh H.L. (2011). Anti-proliferative effects of raw and steamed extracts of Panax notoginseng and its ginsenoside constituents on human liver cancer cells. Chin. Med..

[76] Yan Z., Yang R., Jiang Y., Yang Z., Yang J., Zhao Q., Lu Y. (2011). Induction of apoptosis in human promyelocytic leukemia HL60 cells by panaxynol and panaxydol. Molecules.

[77] Lee C.Y., Hsieh S.L., Hsieh S., Tsai C.C., Hsieh L.C., Kuo Y.H., Wu C.C. (2017). Inhibition of human colorectal cancer metastasis by notoginsenoside R1, an important compound from Panax notoginseng. Oncol. Rep..

[78] Hsieh S.L., Hsieh S., Kuo Y.H., Wang J.J., Wang J.C., Wu C.C. (2016). Effects of Panax notoginseng on the metastasis of human colorectal cancer cells. Am. J. Chin. Med..

[79] Wang C.Z., Luo X., Zhang B., Song W.X., Ni M., Mehendale S., Xie J.T., Aung H.H., He T.C., Yuan C.S. (2007). Notoginseng enhances anti-cancer effect of 5-fluorouracil on human colorectal cancer cells. Cancer Chemother. Pharmacol..

[80] Wang P., Cui J., Du X., Yang Q., Jia C., Xiong M., Yu X., Li L., Wang W., Chen Y. (2014). Panax notoginseng saponins (PNS) inhibits breast cancer metastasis. J. Ethnopharmacol..

[81] He L., Chen X., Zhou M., Zhang D., Yang J., Yang M., Zhou D. (2011). Radix/rhizoma notoginseng extract (sanchitongtshu) for ischemic stroke: A randomized controlled study. Phytomedicine.

[82] Geng H., Zhang L., Xin C., Zhang C., Xie Y. (2022). Xuesaitong oral preparation as adjuvant therapy for treating acute cerebral infarction: A systematic review and meta-analysis of randomized controlled trials. J. Ethnopharmacol..

[83] Zhou D., Cen K., Liu W., Liu F., Liu R., Sun Y., Zhao Y., Chang J., Zhu L. (2021). Xuesaitong exerts long-term neuroprotection for stroke recovery by inhibiting the ROCKII pathway, in vitro and in vivo. J. Ethnopharmacol..

[84] Wu L., Song H., Zhang C., Wang A., Zhang B., Xiong C., Zhuang X., Zang Y., Li C., Fang Q. (2023). Efficacy and safety of Panax notoginseng saponins in the treatment of adults with ischemic stroke in China: A randomized clinical trial. JAMA Netw. Open.

[85] Duan L., Xiong X., Hu J., Liu Y., Wang J. (2018). Efficacy and safety of oral Panax notoginseng saponins for unstable angina patients: A meta-analysis and systematic review. Phytomedicine.

[86] Wang W., Yang L., Song L., Guo M., Li C., Yang B., Wang M., Kou N., Gao J., Qu H. (2021). Combination of Panax notoginseng saponins and aspirin potentiates platelet inhibition with alleviated gastric injury via modulating arachidonic acid metabolism. Biomed. Pharmacother..

[87] Xie Y., Wang C. (2023). Herb-drug interactions between Panax notoginseng or its biologically active compounds and therapeutic drugs: A comprehensive pharmacodynamic and pharmacokinetic review. J. Ethnopharmacol..

[88] Li Y., Lu Y.Y., Jia J., Fang M., Zhao L., Jiang Y., Shi Y., Tu P.F., Guo X.Y. (2021). A novel system for evaluating the inhibition effect of drugs on cytochrome P450 enzymes in vitro based on human-induced hepatocytes (hiHeps). Front. Pharmacol..

[89] Xiao J., Chen D., Lin X.X., Peng S.F., Xiao M.F., Huang W.H., Wang Y.C., Peng J.B., Zhang W., Ouyang D.S. (2016). Screening of drug metabolizing enzymes for the ginsenoside compound K in vitro: An efficient anti-cancer substance originating from Panax Ginseng. PLoS ONE.

[90] Fang Z.Z., Cao Y.F., Hu C.M., Hong M., Sun X.Y., Ge G.B., Liu Y., Zhang Y.Y., Yang L., Sun H.Z. (2013). Structure-inhibition relationship of ginsenosides towards UDP-glucuronosyltransferases (UGTs). Toxicol. Appl. Pharmacol..

[91] Zhang J., Zhou F., Niu F., Lu M., Wu X., Sun J., Wang G. (2012). Stereoselective regulations of P-glycoprotein by ginsenoside Rh2 epimers and the potential mechanisms from the view of pharmacokinetics. PLoS ONE.

[92] Chen X.Y., Qian F., Wang Y.Y., Liu Y., Sun Y., Zha W.B., Hao K., Zhou F., Wang G.J., Zhang J.W. (2021). Ginsenoside 20(S)-Rh2 promotes cellular pharmacokinetics and intracellular antibacterial activity of levofloxacin against Staphylococcus aureus through drug efflux inhibition and subcellular stabilization. Acta Pharmacol. Sin..

[93] Sun M., Zhu C., Long J., Lu C., Pan X., Wu C. (2020). PLGA microsphere-based composite hydrogel for dual delivery of ciprofloxacin and ginsenoside Rh2 to treat *Staphylococcus aureus*-induced skin infections. Drug Deliv..

[94] Zhang J., Sun Y., Wang Y., Lu M., He J., Liu J., Chen Q., Zhang X., Zhou F., Wang G. (2014). Non-antibiotic agent ginsenoside 20(S)-Rh2 enhanced the antibacterial effects of ciprofloxacin in vitro and in vivo as a potential NorA inhibitor. Eur. J. Pharmacol..

[95] Tian Z., Pang H., Du S., Lu Y., Zhang L., Wu H., Guo S., Wang M., Zhang Q. (2017). Effect of Panax notoginseng saponins on the pharmacokinetics of aspirin in rats. J. Chromatogr. B Analyt. Technol. Biomed. Life Sci..

[96] Deng S., Wong C.K.C., Lai H.C., Wong A.S.T. (2017). Ginsenoside-Rb1 targets chemotherapy-resistant ovarian cancer stem cells via simultaneous inhibition of Wnt/β-catenin signaling and epithelial-to-mesenchymal transition. Oncotarget.

[97] Chian S., Zhao Y., Xu M., Yu X., Ke X., Gao R., Yin L. (2019). Ginsenoside Rd reverses cisplatin resistance in non-small-cell lung cancer A549 cells by downregulating the nuclear factor erythroid 2-related factor 2 pathway. Anticancer Drugs.

[98] Liu S., Huang J., Gao F., Yin Z., Zhang R. (2022). Ginsenoside RG1 augments doxorubicin-induced apoptotic cell death in MDA-MB-231 breast cancer cell lines. J. Biochem. Mol. Toxicol..

[99] Zhu C., Wang Y., Liu H., Mu H., Lu Y., Zhang J., Huang J. (2017). Oral administration of Ginsenoside Rg1 prevents cardiac toxicity induced by doxorubicin in mice through anti-apoptosis. Oncotarget.

[100] Wang R.R., Li T., Zhang L., Hu Z.Y., Zhou L., Shan L.T., Huang J.W., Li L. (2023). Acute developmental toxicity of Panax notoginseng in zebrafish larvae. Chin. J. Integr. Med..

[101] Tang J., Zhao M., Li Z. (2020). Immunotoxicity of Panax notoginseng in Sprague-Dawley rats. Chin. J. Public Health.

[102] Zhang K., Sun C., Hu Y., Yang J., Wu C. (2021). Network pharmacology reveals pharmacological effect and mechanism of Panax notoginseng (Burk.) F. H. Chen on reproductive and genetic toxicity in male mice. J. Ethnopharmacol..

[103] Siegel R.K. (1979). Ginseng abuse syndrome. Problems with the panacea. JAMA.

[104] Turck D., Castenmiller J., De Henauw S., Hirsch-Ernst K.I., Kearney J., Maciuk A., Mangelsdorf I., McArdle H.J., Naska A., Pelaez C. (2020). Safety of a botanical extract derived from *Panax notoginseng* and *Astragalus membranaceus* (AstraGin™) as a novel food pursuant to Regulation (EU) 2015/2283. EFSA J..

